# THE ASSOCIATION BETWEEN MEDITERRANEAN DIET ADHERENCE AND ALLOSTATIC LOAD IN OLDER ADULTS

**DOI:** 10.1093/geroni/igac059.1395

**Published:** 2022-12-20

**Authors:** Alexandra Fiocco, Anik Obomsawin, Danielle D'Amico

**Affiliations:** Toronto Metropolitan University, Toronto, Ontario, Canada; X (Ryerson) University, Toronto, Ontario, Canada; Toronto Metropolitan University, York, Ontario, Canada

## Abstract

Allostatic load (AL) is a multisystemic index of biological wear and tear which is associated with poor health outcomes. In recent years, researchers have examined the association between dietary pattern intake and AL; however, no studies to date have examined the relationship between AL and consumption of a Mediterranean diet. Blood and urine samples were collected from 201 community-dwelling older adults who completed a Food Frequency Questionnaire (FFQ). A Mediterranean Diet Score (MDS) was calculated based on previous recommendations and a sex-based AL index was calculated using a count-based approach for 16 biomarkers associated with neuroendocrine, immune, cardiovascular, or metabolic function. It was hypothesized that a higher MDS would associate with lower AL, and that this association would be particularly robust for the immune and metabolic subcomponents of the AL index. In support of the study hypotheses, generalized linear models revealed a significant inverse relationship between MDS and AL (ß = -0.03, P = 0.037). Furthermore, higher MDS was significantly associated with lower immune (ß = -0.06, P = 0.38) and metabolic (ß = -0.05, P = 0.039) subsystem scores, but was not associated with cardiovascular or neuroendocrine subsystem scores. Exploratory analyses further showed that the association was more robust in male than female participants. The current findings are interpreted with caution given the study design and sample characteristics. However, these findings contribute to the literature supporting the Mediterranean diet as an important lifestyle behavior that may support healthy aging.

